# The design evolution of interbody cages in anterior cervical discectomy and fusion: a systematic review

**DOI:** 10.1186/s12891-015-0546-x

**Published:** 2015-04-25

**Authors:** Elizabeth Chong, Matthew H Pelletier, Ralph J Mobbs, William R Walsh

**Affiliations:** 1 University of New South Wales, Randwick, NSW 2031 Australia; 2 Surgical and Orthopaedic Research Laboratory, Prince of Wales Clinical School, UNSW NSW Randwick, 2031 Australia; 3 Neurospine Clinic, Randwick, NSW 2031 Australia; 4 Department of Spine Surgery, Prince of Wales Hospital, Barker St, Sydney, 2031 Australia

**Keywords:** Anterior cervical discectomy fusion, Review, ACDF, Interbody, Cage, Design, Evolution

## Abstract

**Background:**

Anterior cervical discectomy with fusion is a common surgical procedure for patients experiencing pain and/or neurological deficits due to cervical spondylosis. Although iliac crest bone graft remains the gold standard today, the associated morbidity has inspired the search for alternatives, including allograft, synthetic and factor/cell-based grafts; and has further led to a focus on cage fusion technology. Compared to their graft counterparts, cage interbody implants have enhanced biomechanical properties, with designs constantly improving to maximise biocompatibility and osseointegration. We present a systematic review examining the historical progress of implant designs and performance, as well as an update on the currently available designs, and the potential future of cervical interbody implants.

**Methods:**

We performed a systematic review using the keywords “cervical fusion implant design”, with no limits on year of publication. Databases used were PubMed, Medline, Embase and Cochrane. In addition, the search was extended to the reference lists of selected articles.

**Results:**

180 articles were reviewed and 64 articles were eligible for inclusion. Exclusion criteria were based around study design, implant information and patient cohorts. The evolution of cage implant design has been shaped by improved understanding of ideal anatomy, progress in materials research and continuing experimentation of structural design. Originally, designs varied primarily in their choice of structure, however long-term studies have displayed the overall advantages of non-threaded, wedge shaped cages in complementing healthy anatomical profiles, and thus focus has shifted to refining material utilisation and streamlining anterior fixation.

**Conclusions:**

Evolution of design has been dramatic over the past decades; however an ideal cage design has yet to be realised. Current research is focusing on the promotion of osseointegration through bioactiviation of surface materials, as well as streamlining anterior fixation with the introduction of integrated screws and zero profile designs. Future designs will benefit from a combination of these advances in order to achieve ideal disc heights, cervical alignments and fusions.

**Electronic supplementary material:**

The online version of this article (doi:10.1186/s12891-015-0546-x) contains supplementary material, which is available to authorized users.

## Background

Age-related degeneration of the cervical spine is evident in over 50% of the middle-aged population and is the most common cause of neural dysfunction [1]. Although the majority of cases are asymptomatic, changes such as disc herniation, osteophyte formation and hypertrophied ligaments may compress the cervical neuraxis to result in cervical pain, radiculopathy, or myelopathy [2]. First line treatment is conservative; however surgery is indicated in symptomatic patients who are unresponsive to conservative management.

Anterior cervical decompression and fusion (ACDF) is one of the most widely used surgical treatments for patients with cervical spondylosis [3]. It is also an indicated treatment in cases of cervical realignment, trauma and neoplasm [4]. ACDF achieves stabilisation and solid arthrodesis with good-to-excellent clinical outcomes and minimal surgical risks. The anterior approach to cervical decompression was first described by Cloward [5], and Robinson and Smith [6] in the 1950s. Both described an anterior approach via a longitudinal incision along the anterior border of the sternocleidomastoid muscle to allow for soft tissue dissection and annular incision. Following discectomy and removal of any compressive structures, fusion was then achieved using an autogenous graft. Although technical modifications have been made over the years, this procedure is still standard today, leaving improvements in fusion rates and clinical outcomes to be generated through changes in implant design and material [7]. Initially, market available cage materials and designs varied dramatically, with a selection between ceramic and alloy materials in threaded and non-threaded designs. This has shifted dramatically through the years, with modern designs conforming to a non-threaded, wedge shaped profile, and a choice between titanium alloy and the newer, polyetheretherketone (PEEK) materials. This article reviews the evolution of cervical interbody implant designs and assesses future research directions.

## Methods

After performing initial, non-systematic searches using the terms “Anterior Cervical Discectomy Fusion”, “ACDF” and “Cervical Fusion” in conjunction with the terms “cage”, “design” and “implant”, we performed a systematic review of the literature using the following protocol: we searched the databases PubMed, Medline, Embase and Cochrane using the keywords “cervical fusion implant design” for articles available in the English language published up to March 2014. There were no limits or species (See Additional file 1: PRISMA Flow Diagram).

## Results

180 abstracts were searched for relevance and, of these, 64 articles were selected for analysis. Articles were selected based on their detail and relevance to the topic of cage design; both clinical and laboratory studies were included. Laboratory studies comparing cage designs and materials that were controlled and reliable were utilised to inform theoretical advantages of specific designs. The inclusion criteria of clinical studies were prospective and retrospective designss with patients requiring ACDF in the treatment of degenerative cervical disease, patient cohorts larger than 30 individual pati, implanting cages filled with allograft with or without anterior plating. Exclusion criteria included studies with non-degenerative disease cohorts, using additional proteins to promote fusion and ossification, or those that did not report on fusion rates, clinical outcomes and/or complication rates. In addition, the search was extended by manually searching the reference sections of relevant articles; this added 12 publications (See Additional file 2: Table S1).

## Discussion

### Historical evolution: bone grafts to fusion devices

The first anterior cervical interbody techniques were introduced by Cloward, and Robinson and Smith in the 1950s. Cloward’s procedure involved insertion of a dowel graft following decompression. Required bone was harvested from the iliac crest of the patients or via allograft bone bank and was then pre-cut into a graft sized slightly wider and shorter than the drill hole. Insertion was achieved through distraction and force [5]. Robinson and Smith’s approach utilised a similar initial distraction and anterior decompression, however achieved fusion with the insertion of a horseshoe graft harvested from the patient’s iliac crest without the need for extensive graft-site modification.

**Figure 1:**
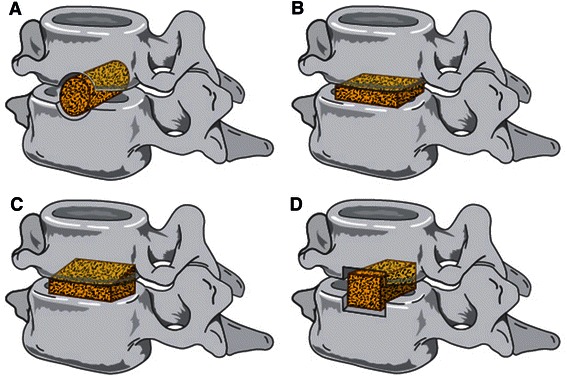
Historical perspectives on ACDF implants. **A)** Cloward Dowel Graft **B)** Smith-Robinson Based Rectangular Implantg **C)** Simmons-Bhalla Keystone **D)** Bailey-Badgley Onlay Strut.

Autograft interbody designs have since evolved to improve stability and distraction. In 1969, Simmons and Bhalla [8] described the benefits of a keystone graft, which increased distraction height by locking into a prepared defect, thereby improving stability and fusion. In 1960, Bailey and Badgley expanded the usage of ACDF to treat neoplasm and instability through the usage of onlay strut grafts. A technique which later evolved into anterior cervical corpectomy [9]. Limitations of the autogenous graft are an important consideration in all of these procedures. Iliac crest bone graft (ICBG) harvesting is associated with high levels of short and long-term morbidity at the harvest site, including pain, wound drainage, infection, haemtomas, nerve injury and iliac crest fractures or deformity [10]. Initially, alternative graft materials were sought as a method of circumventing donor site limitations. However, not only did autograft remain superior in fusion, subsidence and extrusion rates but each alternative involved its own limitations [2,11]. As a result, the focus has switched towards cage implants as a graft substitutes as viable alternatives to autograft (Figure 1).

Cage fusion technology was proposed by Bagby in 1988. Developed to treat spondylotic cervical myelopathy in horses, the Bagby Bone Basket was a cylindrical device made of fenestrated, hollow, stainless steel to allow bone ingrowth into an incompressible spacer [12]. This technology was soon trialled on humans in the lumbar spine and by the 90s was being adapted to the cervical region. Stand-alone designs have since become the mainstay of ACDF, achieving excellent safety, primary stability and long-term fusion results without the limitations and morbidity associated with ICBG options [7,13-15].

### Cage design evolution

The basic design of cages involves a small, hollow implant featuring lateral, upper and/or lower windows to a central cavity filled with either autologous bone, allograft bone or osteoinductive materials [16]. Historically, cage designs can be divided into threaded (screw), and non-threaded cages (vertical rings and box-shaped), with anterior plating applied by surgeon preference. Each type confers individual advantages and disadvantages; an examination of their development allows for insight into future technology. Table 1 contains a comparison of clinical and radiological outcomes of different overall cage types, however it must be taken into consideration that different sample sizes and surgeon cohorts have been included.

**Table 1:** **Clinical and radiological outcomes of different cage designs**

Titanium cage type	Good-to-excellent clinical outcome (%)	Fusion rate at 12 months (%)	Complication rates (%)
Threaded [7,14,66,67]	80-94.4	91-99	11.8-20
Non-Threaded [14,68,69]	75-87	87-95	2-15

### Threaded

#### Screw cages

Screw cages are based on Cloward’s procedure and were some of the earliest available cages. The BAK-C (Sulzer Spinetech, Minneapolis, MN), released in 1994, was a porous, titanium-alloy cylinder packed with surgical site bone-graft [14]. The device was received with considerable success due to its safety and immediate stability, with the advantages of significantly higher stiffness and accelerated fusion when compared to both non-threaded and iliac crest bone graft models [17]. However, studies soon revealed disadvantages of the screw design: decreased maximum distractive height due to the limit on tolerated lateral width of adjacent vertebrae, and higher levels of cage subsidence due to vertebral endplate weakening. In addition, in vitro biomechanical studies comparing screw designs to tricortical bone graft showed screw designs to be less stable during flexion, extension and bending [18,19].

### Non-threaded

#### Box-shaped

Box-Shaped and vertical ring cages resemble Smith-Robinson’s horseshoe graft. Initial designs were rectangular boxes with roughened contact surfaces to improve anchorage [20]. This design demonstrates greater segmental stiffness in all directions compared to both intact segments and tricortical bone grafts, but not compared to their non-threaded counterparts [18]. Further improvements to surface fit have improved cage anchorage through mimicking the inverse shape of the vertebral endplate’s concave contour [21,22]. By early 2000, box-shaped cages began incorporating trapezoidal and wedge-shaped designs [14,16]. Both aim to mimic healthy anatomy of the cervical spine, while increasing segmental stiffness and surface area contact. Trapezoidal cages inversely match vertebral endplates to increase cage stability in lateral bending, flexion and axial rotation [23], whilst wedge-like designs utilise an anterior slope, with a 1-2 mm higher anterior to posterior height, to achieve better restoration of natural cervical lordosis [24,25].

#### Vertical ring designs

In vitro studies have shown little intragroup variation in screw and vertical ring designs, with few differences in their overall advantages and disadvantages; however, one study reported vertical ring designs as having greater control over extension and bending. Compared to intact motion segments, vertical ring designs are reported to have lower rotation stiffness due to the decreased surface area of endplate-implant interface [18].

From a biomechanical perspective, non-threaded cages remain superior to threaded cages due to their ability to mimic healthy cervical anatomy, thereby improving surface contact whilst maintaining initial stability; however new designs are constantly emerging to improve each design by adapting to the natural dimensions of disc space. An early attempt to bridge the difference between a Cloward and Smith-Robinson type design focused on cage dislocation or non-union with instability. The WING cage (Medinorm AG, Germany) attempted a compromise, featuring a cylindrical centre and two lateral flat wings [23]. The cylindrical middle allowed for contact with cancellous bone, with the lateral wings increasing the area of contact with adjacent vertebrae to resist excessive subsidence [23]. Although it achieved good clinical outcomes and fusion rates, the implant was reported to have decreased initial bone contact and primary lateral instability, showing no meaningful advantage over plain screw or box designs [14,26].

The clinical literature comparing different cage shapes is limited, with only one paper reviewing the overall results in a single surgeon cohort [14]. However, some information can be gleaned from following trends in usage, which can be seen to have favoured a non-threaded wedge-shaped, trapezoidal cages; this can be attributed to ease of implantation, greater segmental stiffness and restoration of healthy cervical lordosis.

#### Anterior plating

Stand-alone designs are known to receive good-to-excellent fusion rates with single level ACDF. These results are not achieved in multi-level ACDF, with rates of non-union reported up to 40% in 3-level fusions [27-29]. Anterior plating has been adopted to improve fusion rates and reduce chances of non-union and pseudoarthosis in multi-level ACDF. Anterior metal fixation of bone graft has been employed since 1970, when Schurmann and Busch described the usage of a steel rod reaching adjacent vertebrae [30]. Since then, several anterior stabilisation techniques have been described, with a majority of surgeons using the now standardised technique of Caspar plating [31,32]. Generally, cervical plate fixation improves fusion rates through stabilisation and is associated with improved lordotic alignment, increased disc height, improved fusion rates and lower subsidence rates in both single and multi-level fusions [32,33]. Anterior plating is not without limitations and is associated with additional complications over stand-alone cage procedures, the most common being early postoperative dysphagia, which in rare cases can progress to chronic dysphagia. Other complications include screw migration resulting in soft tissue damage and adjacent-level degeneration in cases of inappropriately sized or misaligned plates [34].

#### Cage materials

Evolution of cage materials has accompanied the changes in design. The field of biomaterials study is widespread and volume constraints dictate that only large trends will be reviewed (see Table 2). Three materials have primarily been used in the manufacture of cage implants: carbon fiber reinforced polymers (CF-P), titanium (Ti) and polyetheretherketone (PEEK). CF-P cages were initially trialled, achieving high rates of fusion and good-to-excellent clinical outcomes however have largely been superseded by PEEK due to its superior elastic modulus [35-38].

**Table 2:** **Clinical and radiological outcomes of different cage materials**

Cage material	Good-to-excellent clinical outcome (%)	Fusion rate at 12 months (%)	Subsidence (%)
CF-P [35,70,71]	76.8	62-98	29.2-49
Titanium [26,42,68,69,72]	46-95	86.5-99	9-45
PEEK [73,74]	80-96	93-100	0-14.2

Ti and its alloys were one of the first materials to be utilised for cages in the 1980s. Used by the orthopaedic world since the 1940s, Ti is a robust biomaterial with excellent corrosion resistance and a low density, that can undergo surface modification to improve osseointegration and cell adhesion [39,40]. PEEK cages were introduced in the 1990s by AcroMed as an alternative to Ti cages; they provide the advantages of radiolucency and an elastic modulus close to bone thereby avoiding the stress shielding associated with Ti [41]. Today, controversy exists between the utilisation of Ti versus PEEK cages. Although PEEK has theoretical advantages, this has not clearly transfered into the clinical setting due to the difficulty in determining and controlling for other surgical factors, including the roles of endplate preparation, area of contact and overdistraction. However, a majority of studies have reported improved fusion rates, lower subsidence rates and radiolucency with PEEK versus Ti cages [42-45], with one long-term study by Chen et al. reporting limited differences in the early postoperative period, but better maintenance of intervertebral height, cervical lordosis and clinical outcomes by PEEK cages in 7-year follow up [46].

### Cage design optimisation

#### Anatomy

Understanding the healthy and pathological anatomy of the cervical spine is vital in optimising the design of cervical cage implants. The first published anatomical studies of the cervical spine in relation to the anterior approach were written in the 90s and have since been quantitatively expanded upon through the use of imaging technology.

Important measurements in reference to ACDF include height, anterior-posterior (AP) diameter and width of the cervical disc space (see Table 3). Disc heights from C2/3 to C6/7 are approximately 1/3 of the vertebral height, with no dependence on age in healthy subjects [47]. In the cervical spine each disc is thinner posteriorly than anteriorly, with the greatest height in the midline, contributing to healthy cervical lordosis, an important consideration in design [48]. Distracted disc height is significantly greater, with the disc space opening nearly 4 mm to accommodate cages of up to 10 mm. The AP diameter increases inferiorly, with shortest depth at C2/3 increasing by 3 mm at C6/7. The lateral width of the disc space also increases inferiorly; in ACDF, implant width is limited by the uncovertebral joint and corresponding endplate concavity.

**Table 3:** **Cervical Disc measurements; measurements were compiled using weighted averages from studies of adult radiographs and cadavers; however a lack of reporting on age, gender and racial variation limits the value of such data [** 47 **,** 48 **,** 51 **,** 58 **,** 75 **,** 76 **]**

	Average disc height*	Anterior disc height (Range)*	Midpoint disc height (Range)*	Posterior disc height (Range)*	Average distracted disc height (Range)	Width of disc space*	Anterior-posterior diameter*	Linear end-plate width
C2/3	3.42	4.07 (3.22-4.92)	4.17 (3.55-4.79)	2.95 (2.09-3.81)	8.5 (6–9)	23.0 (19.0-28.0)	17.9 (13.0-19.0)	15.8
C3/4	3.87	3.42 (2.10-5.52)	4.54 (2.7-5.43)	2.94 (1.70-4.50)	8.8 (6–10)	22.0 (20.0-25.0)	19.8 (17.0-23.0)	17.2
C4/5	4.21	3.28 (1.70-5.32)	4.30 (2.62-4.86)	2.70 (1.66-3.70)	8.8 (8–11)	24.2 (23.0-27.0)	18.8 (16.0-23.0)	17.5
C5/6	3.85	3.30 (2.18-4.92)	4.01 (2.00-4.60)	2.84 (1.4-3.56)	9 (6–13)	25.3 (21.0-30.0)	20.7 (19.0-24.0)	18.5
C6/7	3.37	3.80 (2.79-4.60)	4.63 (2.81-5.3)	2.49 (1.70-3.50)	8.5 (6–11)	28.7 (28.0-35.0)	20.8 (20.0-23.0)	21.8

*columns represent those in which measurements were compiled from weighted averaged.

#### Pathology

**Figure 2:**
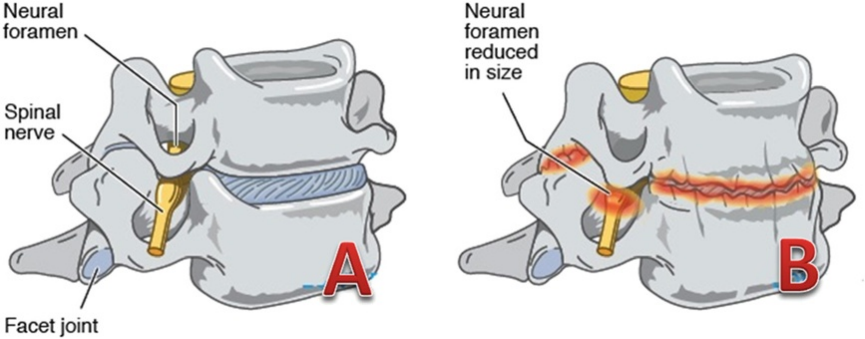
Degenerative Changes of the Cervical Spine. **A)** Healthy cervical vertebrae and disc; **B)** Changes of cervical spondylosis (Disc herniation, osteophyte formation and disc space narrowing leading to reduction in neural foramen size).

In degenerative change, disc space narrowing causes tension in adjacent ligaments and compression of the neuraxis leading to symptomatic cervical spondylosis (Figure 2). The incidence of cervical spondylosis is reported to be as high as 76-82%, however a majority of these individuals are asymptomatic [49]. Classically, cervical spondylosis is believed to involve a combination of nucleus pulposus protrusion, osteophyte formation, and fibrosis, most frequently effecting the C5/6, C6/7 and C4/5 levels, in order of decreasing occurrence [50,51]. Radiographic findings are often poorly correlated to symptomatology; however, visualisation of severe changes, including large osteophyte formation, marked disc space narrowing, sclerosis of vertebral plates and posterior subluxation, are more often associated with pain and discomfort [52]. Macroscopically, there is loss of cervical canal area and foraminal height and area, and flattening of the endplate as the uncinated processes enlarges and flattens to lose its sharp, tapered configuration [53,54].

### Cage dimensions

Modern cage designs have begun targeting individual design features and dimensions to ensure maximal clinical and fusion outcomes. Due to the variation of disc space height between cervical levels and individuals, cage implants are available in a variety of sizes. From surgical experience the author is familiar with variance in only the height of implants, with lateral and AP dimensions remain largely uniform within company models.

#### Cage height

Cervical cages relieve neuraxis compression through the restoration of disc space height, thereby reversing the loss of foraminal height and area, and cervical canal diameter [49,55,56]. The goal of adequate distraction must be tempered by the complications of overdistraction, which is related to non-union, postoperative neck pain and poor clinical outcomes due to an increase in contact pressures between graft and cervical end plates. Originally, Smith-Robinson grafts were recommended to be 10-15 mm in height [6]; however modern grafts are smaller to circumvent the requirement of vertebral modification [57]. In 1993, An et al. demonstrated that ideal distraction height is dependent on baseline disc height, with maximal changes in foraminal height achieved in 2 mm of distraction above baseline [49]. Modern cages adhere to this and available heights ranging between 5-8 mm with trial spacers utilised during surgery to determine ideal cage height.

#### Cage width and length

Cervical implant width and length ensure maximal surface contact and stability of ACDF. These dimensions are dictated by cervical anatomy; too small an implant would provide inadequate stability and too large an implant would result in damage to the surrounding structures [51]. Although lateral disc space width can range between 20-30 mm in the cervical spine, cage implant width is limited laterally by the uncovertebral joint, with ideal placement contacting bilaterally with the uncinated processes [22,58]. Smith-Robinson recommended implants of 14 mm in width and depth, acknowledging the need to modify based on individual requirements [6]. Modern cage designs reflect these dimensions, with lateral widths ranging between 12-20 mm and depths ranging between 12-16 mm [34].

### Modern cage designs

**Figure 3:**
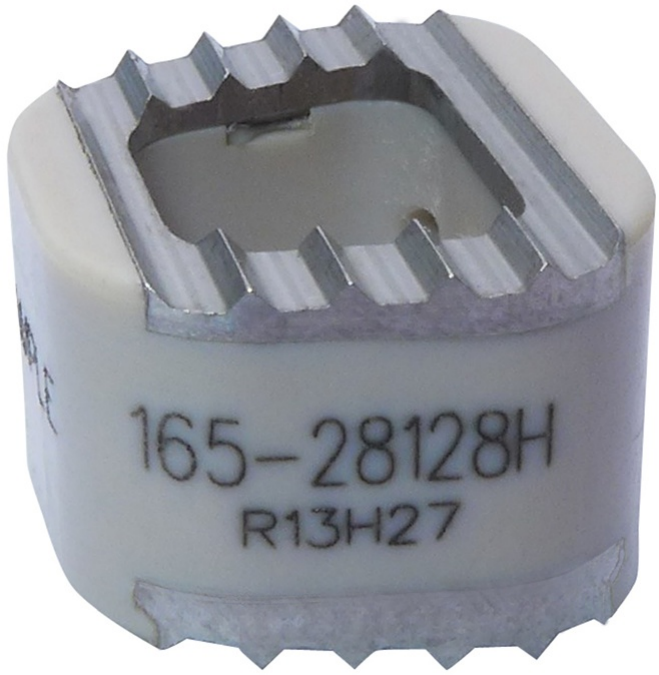
Composite Ti/PEEK Cage. Combo ® cage (A-SPINE Asia, Taiwan) demonstrating ridged titanium endplates on a PEEK interbody spacer.

An ideal cage design would restore healthy alignment and disc height, as well as achieve immediate post-operative stability, high-fusion rates and low complication rates. Recent cage designs have attempted to reduce complication rates by promoting early osseointegration and thus fusion through modification of cage surfaces. Ti and its alloys can be modified to increase surface roughness through plasma beam and electron spray techniques [39]. In vitro experimentation has shown this increases total protein and alkaline phosphatase levels, thereby increasing osteogenic cell differentiation [59]. The improved bioactivity of Ti can be utilised in combination with the elastic modulus and radiolucency of PEEK through the creation of composite Ti/PEEK spacers [60,61]. Clinically available composite spacers, such as the Combo ® cage (A-SPINE Asia, Taiwan), combine PEEK bodies with Ti-endplates (Figure 3) to theoretically augment bone-implant fusion, however there is a dearth in the literature on their comparative efficacy when compared to established clinical and radiographic baselines for Ti or PEEK cages. This requirement for large, long-term clinical studies to verify the relative efficacy of a new cage design is complicated due to the variety of spacers available on the market and the speed at which new designs are released.

**Figure 4:**
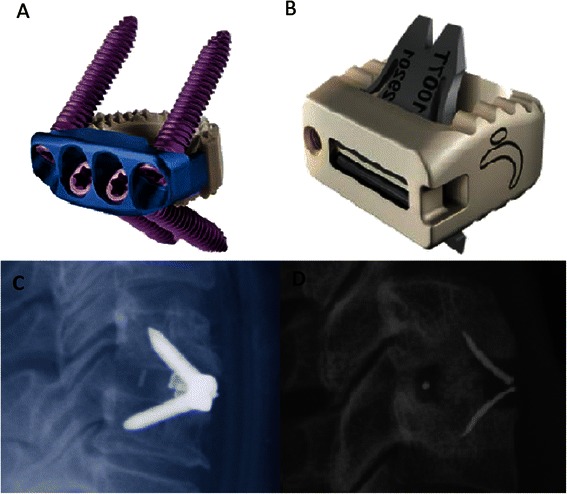
Low Profile Integrated Plating. **A)** Zero-P cervical cage V **B)** ROI-C cervical cage **C)** Radiograph demonstrating Zero-P placement **D)** Radiograph demonstrating ROI-C placement.

Another focus in the improvement of cage designs is the streamlining of anterior plating into a stand-alone cage [62]. Zero-Profile cages utilise an integrated, low profile plate design to avoid implant-to-soft tissue impact, reducing dysphagia rates and other plate-associated complications [34], whilst maintaining good clinical and fusion outcomes [63,64]. Two main designs currently utilise the zero-profile plate system (Figure 4). The Zero-P (Synthes CmbH Switzerland, Oberdorf, Switzerland) was approved by the United States Food and Drug Administration in 2008 and is composed of a PEEK body attached to an anterior plate containing four holes with internal screw treads of either 14 or 16 mm lengths. A second approach to zero-profile plating is adopted by the ROI-C cervical cage (LDR Holding Global Corporation, France), which combines a PEEK body with a self-locking, guided plate system, allowing insertion of plates directly into adjacent vertebrae through the disc space, obviating any need for external hardware. No studies currently exist comparing the efficacy of the two.

**Figure 5:**
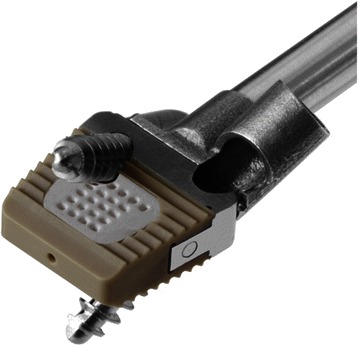
Future Designs. New design integrating a Ti/PEEK composite cage with a low profile plate (Kasios Biomaterials, France).

The combination of integrated low-profile plating and Ti/PEEK composite cages is the next logical step in cage design and is currently undergoing experimental design by Kasios (Kasios Biomaterials, France). The design utilises a PEEK body with titanium endplates and a low-profile titanium plate with dual opposed locking screws (Figure 5).

Recent studies have also explored the development of absorbable designs utilising polylactic acid (PLLA)-polyflycolic acid (PGLA) copolymers and poly(L-lactide-coD,L-lactide), these exhibit the necessary rigidity at the time of implantation with gradual degradation to promote bone formation and solid arthrodesis. In addition, complete postoperative absorption allows improved radiological assessment. However, these benefits are still theoretical, with studies showing high levels of stand-alone cage dislocation requiring revision surgeries [65].

Although the variance in cage design availability has reduced significantly since their first introduction, the amount of research into cage implants has grown. This has trended away from comparing shape designs and fixation to exploring the possibilities posed by the innate material properties, additional growth proteins and the chance for complex 3D printed shapes and streamlined plating designs. Thus although conclusion have been made regarding the optimal cage design in terms of human anatomy, design optimisation needs to become a focus in order to yield the cumulative benefits of each field in an ideal design.

## Conclusion

The evolution of ACDF implant design from bone graft to composite cages has been dramatic; however an ideal implant has yet to emerge. Although there are numerous new designs, difficulties in gathering clinical evidence comparing available models is a limitation in determining the superiority of any one implant. Regardless, trends exist, with shapes favouring wedge-shaped trapezoidal boxes, dimensions reflecting healthy anatomy and a preference towards PEEK bodies. These trends reflect a mixture of clinical evidence and surgical experience, two important factors that continue to influence the ongoing development of ACDF implants. Continued experimentation and integration will be required to achieve further refinement and can be seen in the most recent step of combining bioactive Ti/PEEK composites with the latest zero-profile technology. This paper is not without its limitations, the current search criteria were chosen in order to focus solely on design without performing a full review of all cage related literature. By doing so there will naturally be some articles of relevance not included in the review, however it was determined satisfactory for this article’s purpose.

## Additional files

Additional file 1: Figure S1. PRISMA Flow Diagram.
Additional file 2: Table S1. Additional Articles Reviewed.

## Abbreviations

ACDF: Anterior Cervical Discectomy and Fusion
PEEK: PolyetheretherketoneI
CBG: Iliac Crest Bone Graft
CF-P: Carbon Fibre Reinforced Polymer
Ti: Titanium
AP: Anterior-posterior
