# Supplementary material for: The design evolution of interbody cages in anterior cervical discectomy and fusion: a systematic review
Source: BMC Musculoskelet Disord. 2015 Apr 25;16:99. doi: 10.1186/s12891-015-0546-x (PMC4416390; doi:10.1186/s12891-015-0546-x)
Supplement: Additional file 1: Figure S1. — PRISMA Flow Diagram. [file 12891_2015_546_MOESM1_ESM.doc]

**Additional file 1: Figure S1: PRISMA Flow Diagram**

**Screening**

**Included**

**Eligibility**

**Identification**

Records identified through database searching
(n = 193)

Additional records identified through other sources
(n = 12)

Records after duplicates removed
(n = 180)

Records screened
(n = 180)

Records excluded
(n = 116)

Full-text articles assessed for eligibility
(n = 64)

Full-text articles excluded, with reasons
(n = 12)

Studies included in qualitative synthesis
(n = 64)
