# Supplementary material for: The design evolution of interbody cages in anterior cervical discectomy and fusion: a systematic review
Source: BMC Musculoskelet Disord. 2015 Apr 25;16:99. doi: 10.1186/s12891-015-0546-x (PMC4416390; doi:10.1186/s12891-015-0546-x)
Supplement: Additional file 2: Table S1. — Additional Articles Reviewed. [file 12891_2015_546_MOESM2_ESM.docx]

**Additional file 2: Table S1: Additional Articles Reviewed**

| **Author & Year** | **Type** | **No of Patients** | **Cage Shape (No of Patients)** | **Cage Material** |
| --- | --- | --- | --- | --- |
| Salame et al 2002 | Retrospective Review | 100 | Trapezoidal (100) | Carbon Fibre Reinforced PEEK (100) |
| Van Der Haven et al 2005 | Retrospective Review | 30 | Box Cage (30) | Carbon Fibre Reinforced Polymer (30) |
| Bartels et al 2006 | Retrospective Review | 96 | Wedge-Shaped (96) | Carbon Fibre Reinforced Polymer (96) |
| Hacker 2000 | Prospective Study | 54 | Threaded Cylindrical (54) | Titanium (54) |
| Barlocher et al 2002 | Consecutive Series | 125 | Threaded (36) | Titanium (36) |
| Cauthen et al 2002 | Retrospective Review | 88 | Threaded Cylindrical (88) | Titanium (88) |
| Niu et al 2005 | Retrospective Clinical Study | 54 | Trapezoidal (54) | Titanium (54) |
| Van Jonbergen et al 2005 | Retrospective Clinical Study | 71 | Box Cage (71) | Titanium (71) |
| Matge et al 2002 | Retrospective Review | 250 | Threaded Cylindrical (149)  Wedge-Shaped Impacted Cages (101) | Titanium (191)  PEEK (59) |
| Yang et al 2012 | Retrospective Review | 38 | Horseshoe Box (38) | PEEK (38) |
| Mastronardi et al 2006 | Retrospective Consecutive Series | 36 | Trapezoidal (36) | PEEK (36) |
| Brenke et al 2013 | Prospective Study | 50 | Wedge-Shaped (50) | Absorbable composite cage(Pure β-tricalcium phosphate and Poly DL lactic acid) (50) |

{Matgé, 2002 #4}
